# Guardiola, Klopp, and Pochettino: The Purveyors of What? The Use of Passing Network Analysis to Identify and Compare Coaching Styles in Professional Football

**DOI:** 10.3389/fspor.2021.725554

**Published:** 2021-10-22

**Authors:** Sebastian Immler, Philipp Rappelsberger, Arnold Baca, Juliana Exel

**Affiliations:** Department of Biomechanics, Kinesiology and Computer Science in Sport, Centre for Sport Science and University Sports, University of Vienna, Vienna, Austria

**Keywords:** coaching, football (soccer), notational analysis, social network analysis, collective behavior

## Abstract

We applied social networks analysis to objectively discriminate and describe interpersonal interaction dynamics of players across different top-coaching styles. The aim was to compare metrics in the passing networks of Jürgen Klopp, Pep Guardiola, and Mauricio Pochettino across the UEFA Champions League seasons from 2017 to 2020. Data on completed passes from 92 games were gathered and average passing networks metrics were computed. We were not only able to find the foundations on which these elite coaches build the passing dynamics in their respective teams, but also to determine important differences that represent their particular coaching signatures. The local cluster coefficient was the only metric not significantly different between coaches. Still, we found higher average shortest-path length for Guardiola's network (mean ± std = 3.00 ± 0.45 a.u.) compared to Klopp's (2.80 ± 0.52 a.u., *p* = 0.04) and Pochettino's (2.70 ± 0.39 a.u., *p* = 0.01). Density was higher for Guardiola's (64.16 ± 20.27 a.u.) than for Pochettino's team (51.42 ± 17.28 a.u., *p* = 0.008). The largest eigenvalue for Guardiola's team (65.95 ± 16.79 a.u.) was higher than for Klopp's (47.06 ± 17.25 a.u., *p* < 0.001) and Pochettino's (42,62 ± 12.01 a.u., *p* < 0.001). Centrality dispersion was also higher for Guardiola (0.14 ± 0.02 a.u.) when compared to Klopp (0.12 ± 0.03 a.u., *p* = 0.008). The local cluster coefficient seems to build the foundation for passing work, however, cohesion characteristics among players in the three teams of the top coaches seems to characterize their own footprint regarding passing dynamics. Guardiola stands out by the high number of passes and the enhanced connection of the most important players in the network. Klopp and Pochettino showed important similarities, which are associated to preferences toward more flexibility of interpersonal linkages synergies.

## Introduction

Although the game model is a process dependent on a diversity of interacting constraints that go beyond the ideas of coaches (Ribeiro et al., 2019a), coaches are the ones responsible for defining the tactical principles under which teams will pursue the intended performance outcomes during attacking and defending plays (Garganta et al., 1997). The self-organization and actions of players will be constantly updated according to the context of the performance environment (i.e., behavior and score of opponents), and the best solution or the most appropriate tactical pattern of play can be performed to achieve the intended outcome. Yet, the elaboration of the strategies underlying the collective behavior of a team is part of coach assignments.

Since tactical principles influence the collective organization of a team (Clemente and Martins, 2017), it might be reasonable to associate ball passing information as an important outcome of teammates interaction, especially related to interpersonal coordination during attacking building (Aquino et al., 2020). Previous studies have identified different interaction levels among players according to their respective playing position, both in 3rd Brazilian Division (Aquino et al., 2020) and in German Bundesliga (Korte et al., 2019). In the Bundesliga, the pass outcome was also accounted and showed that central defenders would function more often as intermediary players and were present in most of the unsuccessful plays while in successful plays, the offensive midfielders were most involved and the defensive midfielders were the main intermediary players. Forwards, although not identified as playmakers as the external midfielders, were most frequently involved in successful plays. These are practical and important examples of contributions of individual players to match interplays and of pattern identification in the passing structure of sports teams.

The patterns of interaction between players through ball-passing can also assess performance at its macro or collective level. Because sports teams can be conceptualized as complex social networks (Passos et al., 2011), the interaction of the different system agents (players) and further self-organizing behaviors during team coordination can be measured (Ribeiro et al., 2019b), and structural and topological properties can be taken. The quantification of these properties is achieved through the computation of the connectivity profiles associated between the nodes (representing the players) linked by edges (representing a performance marker, as completed passes), thus, allowing for understanding the way these elements are embedded in the network. As such, influences of the match scoring on team playing behavior have already been identified, for example. Mostly, scoring status (Praça et al., 2019) and the magnitudes of the final score of match sequences (Clemente et al., 2019) alter the level of participation of playing positions during passing. Other contexts have also been modeled by social network analysis (SNA). The venue and quality of opposition on different styles have been reported to produce a change in the average use of attacking and defensive style of play in association football (García-Rubio et al., 2015).

It is therefore possible that the assessment of the emergent interpersonal interactions between teammates under the constraints of competitive environments (Ribeiro et al., 2017), and patterns regarding the collective organization during matches (Gyarmati et al., 2014) may possibly acknowledge the playing style of coaches. Recently, the signature of the Barcelona era of Guardiola was remarkably unveiled through the application of SNA metrics in data from the Spanish national league in the 2009/2010 season (Buldú et al., 2019). Authors could identify how the organization of Barcelona under the coaching of Guardiola differed from the opponents in all of the most important variables.

The Best FIFA Men's Coach is an award given after a voting process in which football fans, selected media representatives, captains, and head coaches of national teams at a global level participate. In 2019, the 3 finalists were Jürgen Klopp, Pep Guardiola, and Mauricio Pochettino. These coaches, which have had relatively long careers in the supervision of Liverpool, Manchester City and Tottenham Hotspur teams, respectively, also particularly had a successful campaign in the Champions League that year. Such recognition of an outstanding work by interested people and experts involved in football association brings curiosity over the possibilities of measuring their gameplay footprints, thus going beyond impressions. In this study, we explored the potential of passing social networks to objectively discriminate match dynamics across different coaching framework styles. Therefore, the aim was to quantify similarities and differences in the passing networks of Jürgen Klopp, Pep Guardiola, and Mauricio Pochettino across the UEFA Champions League seasons from 2017 to 2020. We hypothesize that passing network metrics will show particular patterns on the work of each coach and, timewise, slight modifications in team performance will be revealed, especially comparing to the 2018/19 season, in which they were the finalists in the Best FIFA Men's Coach award.

## Materials and Methods

### Sample

In total, passing data on 92 games of the UEFA Champions League were analyzed. These were all games played by the teams coached by Jürgen Klopp (*n* = 34 games), Pep Guardiola (*n* = 29), and Mauricio Pochettino (*n* = 29) during the seasons of 2017/18, 2018/19 and 2019/20, while coaching Liverpool, Manchester City, and Tottenham Hotspur teams, respectively. In the 2017/18 season, Klopp's team reached the finals (*n* = 13 games), Guardiola's team reached the quarter finals (*n* = 10) and Pochettino's team reached the round of 16 (*n* = 8). In the 2018/19 season, Klopp's team reached the finals (*n* = 13), Guardiola's team reached the quarter finals (*n* = 10) and Pochettino's team reached the finals (*n* = 13). In the 2019/20 season, Klopp's team reached the round of 16 (*n* = 8), Guardiola's team reached the quarter finals (*n* = 9 since no rematch was played due to COVID-19 pandemic), and Pochettino's team reached the round of 16 (*n* = 8). Data from the games were gathered from technical reports contained in the media press kits of UEFA, which were published after every game and are available online (UEFA, 2021).

### Social Network Analysis

A dedicated toolbox for social network analysis (MIT, 2011) was applied, and the passing data from the 11 players that played most time during the matches were included. In graph approach of network analysis, the nodes are the individual players and the links represent the number of passes between them. The links are unidirectional and weighted according to the number of passes between players. The network metrics calculated for the present study were the following: local clustering coefficient, density, average shortest-path length, mean centrality, and largest eigenvalue. For each match, all variables were calculated from adjacent matrices normalized by the maximum number of passes performed to avoid the bias of a different number of passes in each game to impact the interpretation of the results. As already described previously (Ramos et al., 2018; Buldú et al., 2019), the local clustering coefficient was calculated as the all-players average of the probability that neighbors of a given player in the team will also be connected between them. This way, in a weighted network (the number of passes between players is not the same), not only the number of nodes connected between them are accounted but, also, how the link weights are distributed. It provides a measure of local robustness by relating to the tendency of a team to form balanced triangles between players. Density is an indicator of cohesion between network members. When the density gets smaller, the likelihood of the network being split into independent groups instead of one interconnected network, increases. The average shortest-path length describes the smallest number of links (passes) needed to connect two nodes (players) in a network. For weighted passing networks, the topological distance between nodes are calculated as the inverse of the number of passes between players, so the higher the weight, the shorter the distance between two nodes. The largest eigenvalue is a measure of the strength of a network and is dependent on the number of passes and which players are involved. The higher the number of passes and involvement of the most important players in these passes, the higher the largest eigenvalue. The components of the eigenvector for each node that corresponds to the greatest eigenvalue was obtained as the centrality scores of the players in the network. Additionally, the dispersion of the centrality was calculated and, as an indicator of heterogeneity in the network structure, would display how (un)evenly the importance of each player in the network is distributed.

### Data Analysis

Univariate statistics were applied to the data to compare the overall passing network metrics between the three coaches, and to compare coaches individually across seasons. The Shapiro-Wilk-Test was used to test the normality of the data. For the normally distributed data, one-way ANOVA with LSD *post hoc* were carried out to check for statistical significance in the differences. For the not normally distributed data, Kruskal-Wallis H test with Dunn-Bonferroni *post hoc* were carried out. The Epsilon-squared estimate of effect size for the Kruskal-Wallis test, using adjusted H values were calculated, and the following categories defined small (0.01), medium (0.06), and large (0.14) effects (Levine and Hullett, 2002). The level of significance was considered as *p* < 0.05.

To test the grouping behavior in the association of such a multivariate profile of SNA metrics, we associated Principal Component Analysis (PCA) with K-means clustering technique. It consisted of the calculation of eigenvalues and eigenvectors from the covariance matrix of the dataset. Data from each variable was previously normalized by its corresponding variance to minimize the effect of discrepancy in the values of the SNA metrics. The eigenvalues are coefficients corresponding to the variances of each component, which represent the loading factors of the original data to the calculation of the new transformed dataset. The scores were calculated by multiplying the original dataset and centered in the mean by the eigenvectors. The eigenvectors are the vectors containing these coefficients (or eigenvalues), and, after being ranked in order of its eigenvalues, the principal components in order of significance were found. Because the 1st principal component explained most of the variance of the new transformed dataset (98.4%), it was the only one selected for further analysis. Then, we applied k-means clustering to partition the scores of the 1st principal component into three groups. To get an idea of how well the resulting clusters were separated, we calculated the mean silhouette coefficient for the scores associated with each cluster group. This measure ranges from +1, indicating scores are very distant from neighboring clusters, to −1, indicating scores are probably assigned to the wrong cluster. In previous literature, values between 0.71 and 1.00 are stated as strong, 0.51 and 0.70 as reasonable, 0.26 and −0.50 as weak, and ≤0.50 as non-substantial structures to be found (Kaufman and Rousseeuw, 1990).

## Results

### Overall Social Network Metrics

The passing network metrics results are described as median and confidence intervals in Table 1. The Kruskall-Wallis test showed differences between the coaches in density [$\chi_{\left(2\right)}^{2}$ = 7.01, η^2^ = 0.08, *p* = 0.03], average shortest-path length [$\chi_{\left(2\right)}^{2}$ = 7.08, η^2^ = 0.08, *p* = 0.03], and mean centrality [$\chi_{\left(2\right)}^{2}$ = 7.12, η^2^ = 0.08, *p* = 0.03]. The one-way ANOVA revealed that the algebraic connectivity [*F*_(2,89)_ = 18.42, *p* < 0.001]. The overall local cluster coefficient did not present any significant difference among coaches.

**Table 1:** Passing network metrics descriptive of Klopp's, Guardiola's and Pochettino respective teams (Liverpool FC, Manchester City FC, and Tottenham Hotspur FC along 3 UEFA Champions League seasons).

**Coach**	**Season**	**Density**	**Local clustering**	**Average shortest-**	**Centrality**	**Largest**
			**coefficient**	**path length**	**dispersion**	**eigenvalue**
		**Median [C.I.]**	**Median [C.I.]**	**Median [C.I.]**	**Median [C.I.]**	**Median [C.I.]**
**Jüngen Klopp**	2017/18	50.49 [40.39–78.19]	0.43 [0.41–0.65]	2.47 [2.32–3.04]^c^	0.11 [0.10–0.13]	34.03 [31.53–56.07]^c^
	2018/19	50.54 [40.11–59.99]	0.44 [0.40–0.68]	2.56 [2.43–2.99]^c^	0.13 [0.10–0.14]	41.45 [33.60–50.61]^c^
	2019/20	59.97 [47.71–85.26]	0.46 [0.37–0.66]	3.01 [2.79–3.45]	0.13 [0.10–0.14]	60.36 [52.52–68.26]
	**Overall**	**53.60 [48.88–66.03]**	**0.45 [0.46–0.60]** ^a^	**2.82 [2.61–2.98]**	**0.13 [0.11–0.13]** ^a^	**44.47 [41.04–53.08]** ^a^
**Pep Guardiola**	2017/18	70.50 [56.71–89.66]	0.44 [0.42–0.46]	2.82 [2.59–3.41]	0.15 [0.14–0.16]	73.03 [60.04–86.11]
	2018/19	53.35 [46.21–62.33]	0.45 [0.42–0.76]	2.81 [2.61–3.08]	0.14 [0.13–0.15]^b^	56.18 [51.88–71.10]
	2019/20	56.72 [48.38–81.84]	0.45 [0.37–0.74]	3.25 [2.88–3.48]	0.12 [0.11–0.13]^b^	58.84 [49.40–76.54]
	**Overall**	**56.77 [56.45–71.87]**	**0.45 [0.45–0.60]**	**2.95 [2.83–3.17]**	**0.14 [0.13–0.15]**	**58.84 [59.56–72.33]**
**Mauricio Pochettino**	2017/18	52.85 [40.67–71.05]	0.43 [0.35–0.75]	2.55 [2.28–2.92]	0.13 [0.09–0.15]	44.61 [33.13–54.60]
	2018/19	43.35 [41.11–62.10]	0.42 [0.39–0.66]	2.58 [2.43–2.96]	0.13 [0.11–0.15]	43.18 [35.14–49.24]
	2019/20	44.96 [32.23–61.10]	0.42 [0.33–0.74]	2.91 [2.53–3.07]	0.14 [0.11–0.16]	43.55 [30.97–53.18]
	**Overall**	**45.10 [44.85–57.99]** ^a^	**0.42 [0.45–0.62]** ^a^	**2.62 [2.55–2.85]**	**0.13 [0.12–0.14]**	**43.28 [38.05–47.19]** ^a^

a *Significantly different from overall Guardiola (p < 0.05);*

b *significantly different from season 2017/18;*

c *significantly different from season 2019/20. Bold values indicate the overall values of the 3 seasons considered in this study*.

The Kruskall-Wallis multicomparison showed significant higher density for Guardiola's network (mean ± std = 64.16 ± 20.27 a.u.; mean rank = 18.50) compared to Pochettino's (51.42 ± 17.28 a.u., *p* = 0.008). He also showed higher average shortest-path length for his network (3.00 ± 0.45 a.u.) than Klopp (2.80 ± 0.52 a.u.; 17.67; *p* = 0.01) and Pochettino (2.70 ± 0.39 a.u.; 13.76; *p* = 0.04). Mean centrality was also higher for Guardiola's team passing network (0.14 ± 0.02 a.u.) compared to Klopp's (0.12 ± 0.03 a.u.; 17.78; *p* = 0.008). Additionally, The LSD *post-hoc* from one-way ANOVA showed that the largest eigenvalue was higher for Pep Guardiola's network (65.95 ± 16.79) compared to Klopp's (47.06 ± 17.25; *p* < 0.001) and Pochettino's (42.62 ± 12.01; *p* < 0.001).

### Seasonal Social Network Metrics

The comparisons between the network of Jürgen Klopp across the 3 seasons showed significant differences for the average shortest-path length [$\chi_{\left(2\right)}^{2}$ = 7.32, η^2^ = 0.22, *p* = 0.02] and largest eigenvalue [*F*_(2,31)_ = 3.66, *p* = 0.04]. The 2019/20 (3.12 ± 0.40 a.u.) season showed higher average shortest-path length when compared to 2017/18 (2.68 ± 0.59 a.u.; 11.78; *p* = 0.008) and 2018/19 (2.71 ± 0.46 a.u.; 9.47; *p* = 0.03). The largest eigenvalue also was higher in the latest season (66.49 ± 22.46 a.u.) than 2017/18 (59.29 ± 31.28 a.u.; *p* = 0.03) and 2018/19 (50.05 ± 16.45 a.u.; *p* = 0.02).

Pep Guardiola passing network had a higher mean centrality in 2019/20 (0.13 ± 0.03 a.u.) when compared to the 2017/2018 season (0.12 ± 0.04 a.u.; *p* = 0.001). No other significant differences were found.

No significant differences across seasons were found for the passing network metrics of Pochettino.

### Principal Component and Cluster Analysis

**Figure 1:**
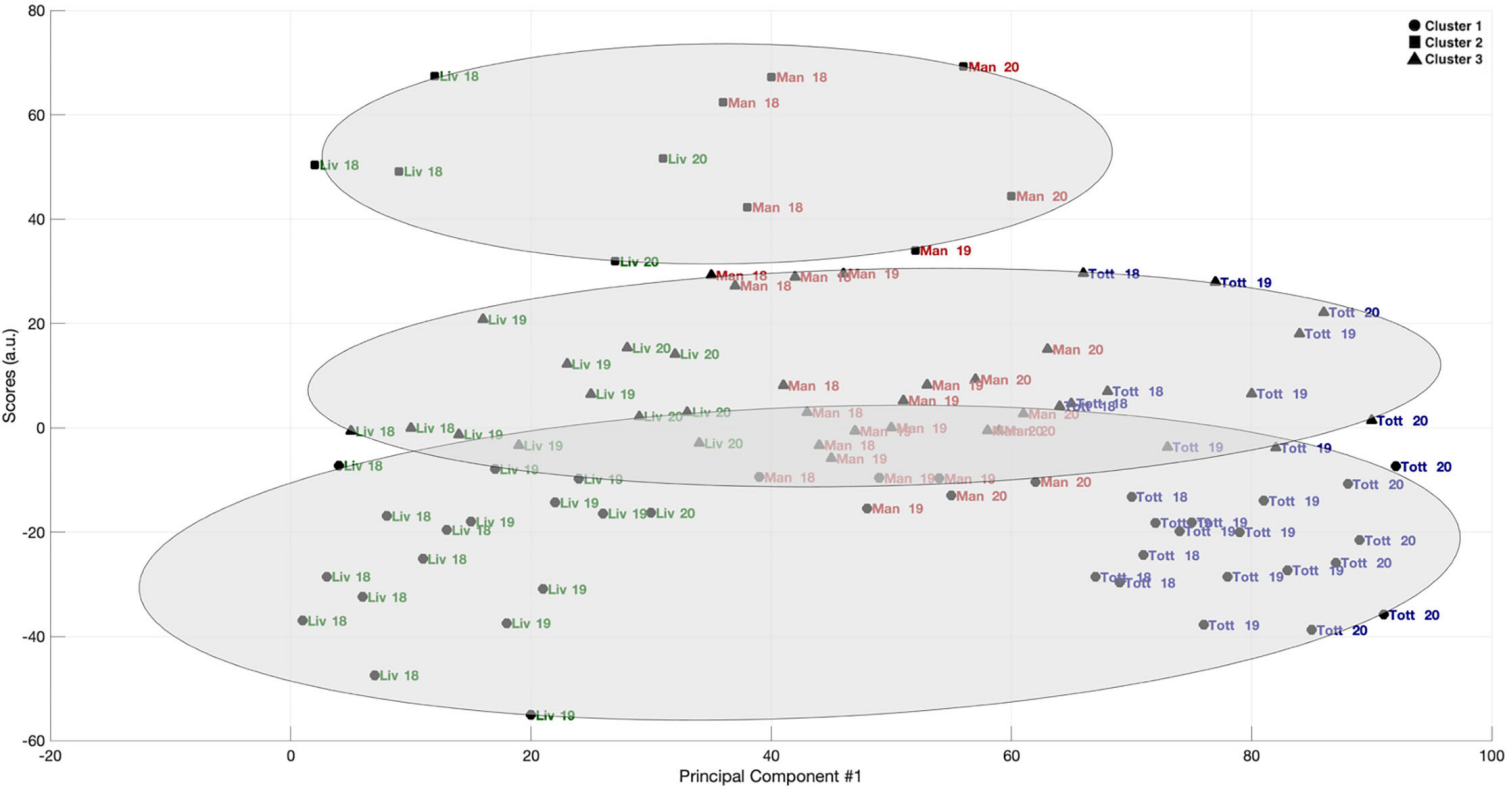
Principal component scores and clustering related to the passing network metrics from Liverpool FC. Manchester FC and Tottenham Hotspur in the 2017/18, 2018/19, and 2019/20 seasons of the UEFA Champions League, coached by Jürgen Klopp, Pep Guardiola and Mauricio Pochettino, respectively.

**Figure 2:**
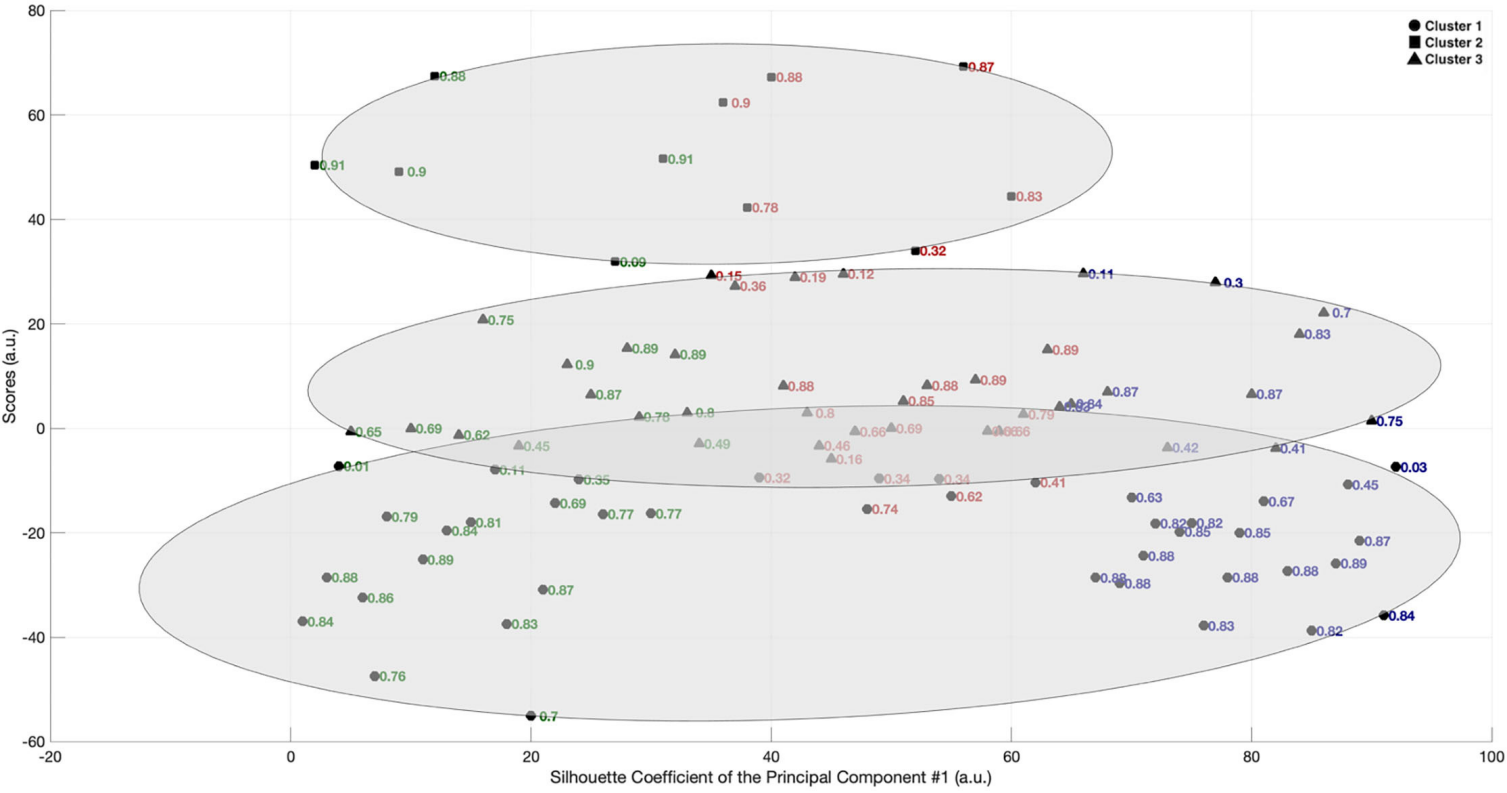
Clustered principal component scores and correspondent silhouette coefficients related to the passing network metrics from Liverpool FC (green), Manchester FC (red) and Tottenham Hotspur (blue) in the 2017/18, 2018/19, and 2019/20 seasons of the UEFA Champions League, coached by Jürgen Klopp, Pep Guardiola and Mauricio Pochettino, respectively.

The eigenvectors from the covariance matrix of the network variables are presented in Table 2. From the five eigenvalues, the ones related to the first principal component were found to explain 88% of the variance of the total dataset. The coefficients referent to the first principal component are also presented in Table 2 and can be stated as the “weight” of the original passing network variables in the PCA, referring to the relative importance of each variable. Density and local cluster coefficient showed the highest coefficients for the first principal component, therefore, the scores for the cluster analysis were obtained using only the first principal component. K-means clustering was applied to classify this new data set into three groups, with the results being presented in Figures 1, 2. Figure 1 shows how the teams were classified in each cluster. The scores were labeled according to the correspondent team and season. Figure 2 represents cluster classification as well, but with the scores labeled according to each individual silhouette coefficient obtained. The mean silhouette coefficients were 0.70 for Cluster 1, 0.80 for Cluster 2, and 0.60 for Cluster 3. Most scores associated to the passing network of Klopp and Pochettino were classified in the Cluster 1 (50 and 62% of the scores, respectively). Most of the passing network scores of Guardiola were classified in Cluster 3 (59%).

**Table 2:** Total variance explained by the first principal component and loadings of its corresponding eigenvectors.

**Variance explained**	**%**	**Eigenvectors**	
Principal component #1	88.0	Density	706.7
		Local clustering coefficient	94.6
		Average shortest-path length	0.12
		Mean centrality	0.04
		Largest eigenvalue	0.00

## Discussion

The primary objective of this study was to identify the match dynamics across the different coaching frameworks of Jürgen Klopp, Pep Guardiola, and Mauricio Pochettino while supervising the FC Liverpool, Manchester City FC, and FC Tottenham Hotspur during three seasons, through passing network analysis. In fact, we were not only able to find the foundations on which these elite coaches build the passing dynamics in their respective teams, but also to determine important differences that might represent their particular coaching styles. We also found that these coaches presented changes in their framework following the 2018/2019 season, in which they were nominated to the Best FIFA Men's Coach awards.

### Overall Results

According to the results of the univariate analysis, overall, the local clustering coefficient seem to build the foundation of passing dynamics of coaches, since it was similar among them. Indeed, it is an indicator that has been previously associated with success in football coaching at different levels of performance (Buldú et al., 2019). Previous research has reported that clustering coefficients of world champions are higher when compared to other teams, as in the case of Spain in the FIFA 2010 World Cup (Peña and Touchette, 2012) and Germany in 2014 (Clemente et al., 2015). At the European level, previous analyses on elite teams during UEFA Champions League 2015/2016 found evidences of higher values for teams in the quarter finals compared to other stages from the competition (Clemente and Martins, 2017). However, similarities have been found, also at this level, in network metrics as clustering/cohesion for goal-scoring plays at any competition stage and match status (Mclean et al., 2018). The local clustering coefficient indicates the capacity of players to cluster in communities of triplets or to unite the team, thus relating to cooperation processes (Clemente and Martins, 2017; Mclean et al., 2018). The present results on this variable reveal that, overall, the three top coaches promote similar engagement between subgroups of players to coordinate actions during matches. Our results may indicate that the feature of passing dynamics that represents cooperation and coordination properties, as the local clustering coefficient, is a baseline for coaches' work at European-league level.

Despite this, Guardiola seems to have a proper signature to his work in terms of the passing dynamics output by Manchester City. His team presented higher values for all the remaining network metrics. When compared to the passing network of both Klopp and Pochettino, Pep Guardiola's showed higher largest eigenvalue and average shortest-path length. Higher largest eigenvalue overall indicates that the passing network of Guardiola had a higher number of passes than Klopp's and Pochettino's. Moreover, better passing connections between the most important players in the network may increase the largest eigenvalue as well. Previous studies have shown the largest eigenvalue to be associated to the network strength and less susceptibility to errors (Aguirre et al., 2013; Buldú et al., 2019). In other words, the negative impact of incomplete passing on Manchester City would be likely smaller, since the present results might indicate that players demonstrated enhanced ability in exploring passing options, thus evidencing a higher synergy in team coordination. Buldú et al. (2019) described the same for Guardiola's work during Barcelona's campaign in La Liga 2009/2010 (the season in which he won the titles of six major competitions, including the UEFA Champions League). Non-academic literature has already pointed out ball possession as part of his philosophy (Perarnau, 2016), however, the current study successfully quantifies that his coaching style is indeed focused on ball possession and team coordination. The higher average shortest-path length found for Guardiola's network indicates that the pairs of players in his team are less closely connected, which might lead to the participation of more intermediate players to connect such passes. This result contrasts with what was previously reported for Guardiola when coaching the FC Barcelona during La Liga 2009/10 (Buldú et al., 2019). Maybe this is a feature that compensates for the higher largest eigenvalue. Thus, to be able to explore options successfully, the participation of a higher number of players in the passing might have been the solution to maintain proper team synergy levels.

On the other hand, presenting a smaller topological distance between pairs of players might give a team more options to distribute the ball. This can be beneficial during long ball possessions or when transitioning to offense after recovering the ball. Thus, the results for the average shortest-path length for Jürgen Klopp and Mauricio Pochettino might explain some strategies adopted during matches. Changes in player positions during the game (i.e., full backs switching sides for a possession after a corner play on the respective other side, or wingers switching sides from time to time) would potentially lead to higher proximity of players to different teammates during the course of a game. Therefore, changing tactical patterns of play can alter the topological distances of players regarding passing dynamics. It can be argued then that compared to Guardiola, the coaching style of Klopp and Pochettino might favor the degeneracy property of synergy coordination in his team. Team performance is dependent on the capacity of players in exploring and mutually adapting to teammates and opponent behavior variability (Araújo and Davids, 2016). A functional movement variability reflects the emergency of degeneracy, which is fundamental to yield a flexible and robust performance (Dodel et al., 2020). Thus, this result might also reflect increased adaptability and flexibility in the collaborative behavior of Liverpool FC and Tottenham Hotspur players, in order to satisfy the game constraints and maintaining passing dynamics performance.

Density only showed to be different between the passing networks of Pep Guardiola and Mauricio Pochettino. This is a measure of structural cohesion inside a team and has been reported as a predictor of success in offensive plays in UEFA Champions League games (Pina et al., 2017) and the FIFA World Cup 2014 (Clemente et al., 2015). Increased density values, as found for Guardiola, are associated to a higher number of passes, longer ball-possession periods and fewer loss of possession (Pina et al., 2017). Guardiola particularly achieves a higher level of interconnection in the passing structure of his teams when compared to Pochettino. However, it is interesting to observe that from clustering and cohesion-related measures, the pattern of distribution of such cooperative interactions among teammates is more unbalanced in the team of Mauricio Pochettino once he presents low values. Then, this result might relate to the features of Tottenham Hotspur interpersonal linkage that were discussed by Ingold (2015) and Araújo and Davids (2016). He might favor players ability to move in synergy, as a functional unit, and yet, allow them opportunities to contribute to the team with their individual skills. However, it does not necessarily affect other important aspects of the team passing dynamics.

Pep Guardiola showed higher centrality dispersion when compared to Jürgen Klopp. This particular metric describes the distribution of the levels of importance of players inside the passing network, thus it seems that Klopp's style while coaching FC Liverpool relied less in particular players ability to hub the team network than Guardiola's. This result is in accordance with data previously reported for his past work as the coach of Barcelona. Literature has reported that at high performance levels, i.e., World Cups, successful teams tend to present a no-star topology feature in their passing networks (Grund, 2012; Cotta et al., 2013). The present results suggest that in high-level football association, teams presenting lower centrality, or lower dependency on particular players, is not a consensus among successful coaches.

Although the passing networks of Jürgen Klopp and Mauricio Pochettino had specific disparities with Pep Guardiola's, both showed important similarities in the dynamics of their networks. The multivariate and grouping analysis supports these overall results, by giving a reduced dimension on what assigns to coaching styles. Most of the data variance, when linearly combined to the other network metrics, was explained by the first principal component that showed to be mostly influenced by the density and local clustering coefficient, respectively. While the first seems to define how interconnected the players are inside a team as a signature and separating factor, as for the team of Guardiola, the latter is the one to show the common ground between the three coaches. The grouping analysis had a high degree of separation, with mean silhouette coefficients >0.6, and showed around 62% of Pochettino's and 50% of Klopp's network scores classified in Cluster 1, while 59% of Guardiola's scores were classified in Cluster 3. Thus, our results may confirm that the features of passing dynamics which represent cooperation and coordination properties assign marks to coaches' work in high-level football association.

### Coaches Across Seasons

It was in the 2018/19 season when all three coaches were nominated for The Best FIFA Men's Coach award. Jürgen Klopp received the award (altogether with his team winning the Champions League) and was also the winner in the following season. The present results revealed slight but remarkable changes in the works of the three nominees across time, especially in the season after the nomination.

The network average shortest-path length and largest eigenvalue of Klopp changed from the 2019/20 in comparison to the previous seasons. These variables increased along seasons, indicating an attempt to balance the increase of the topological distances between players connected through passes with an improvement of the general cohesion between the players, including important players in the hub to enhance such connection.

The network of Pep Guardiola showed a decrease in the centrality dispersion across seasons. Thus, this may be the strategy to balance and become the higher mean centrality more functional, which is also characteristic of his network metrics. These results might also reflect a change in the quality of players between seasons. It has been reported that Guardiola assigns central roles of his passing networks dynamics to particular players (Buldú et al., 2019). It is possible to speculate that maybe player transfers during the seasons could be linked with the changes in the importance of players inside the network. Reports show transfers/(returning from) loans in and out of Manchester in 2017/18 were 26/28 respectively, and decreased in the following seasons, with 17/16 in 2019/19, and 18/20 in 2019/20 (Transfermarkt, 2020). One might argue that part of this phenomenon can be associated to the capacity of Guardiola to fine-tune the important roles amongst his players, thus reducing the need of acquiring new players.

In the seasons 2017/18 and 2019/20, Tottenham FC dropped out of the Champions League in the quarter finals, while they reached the finals in 2018/19, losing against the FC Liverpool of Klopp. For the better or for the worse in terms of performance, Pochettino is the coach whose passing network showed no difference in its metrics across seasons. He was mostly classified in Cluster 1, along Klopp, but presented the lowest values for most of the network metrics that did not change across time. It is worth to highlight that the average local clustering coefficient did not change for any of the coaches across the seasons, reinforcing the idea that these two metrics play a fundamental role in overall coaching.

There are some limitations regarding this study that should be addressed accordingly. The present study only considered players who played most time in the matches, and such limitation can affect the topological properties of the network as substitutions may lead to lower number of passes. This choice was made to avoid misleading parameters referred to players (i.e., as centrality dispersion). Also, although it was possible to characterize passing dynamics framework of three top-coaches in high-level European football association teams over three seasons, we did not have access to positional data of players during the matches. Thus, a spatial-temporal analysis associated to the passing dynamics was not possible, but would contribute to reveal the coordinative behaviors underlying the passing outcome at both dyads and team levels.

## Conclusion

The present study characterized three top-coaches style regarding passing dynamics, while coaching the same teams during three consecutive seasons. It was also possible to find their common ground and their work signatures through social network analysis of passing data. The local clustering coefficient seems to build the foundation for passing work, therefore, promoting engagement between subgroups of players to coordinate actions during matches is key for coaches. However, Pep Guardiola has his own footprint regarding passing dynamics and relevant players in connecting attacking plays and maintaining that important interaction between players seems to define it. Thus, Guardiola stands out by the coordinated and integrated connection between the communities of players. Klopp and Pochettino share most similarities in their style. Both seem to explore more the flexibility of interpersonal linkages synergies, with fluid opportunities or level of collaboration between all teammates.

## Data Availability Statement

Publicly available datasets were analyzed in this study. This data can be found at: https://www.uefa.com/insideuefa/mediaservices/presskits/index.html.

## Ethics Statement

Ethical review and approval was not required for the study on human participants in accordance with the local legislation and institutional requirements. Written informed consent for participation was not required for this study in accordance with the national legislation and the institutional requirements.

## Author Contributions

SI: data collection, data analysis, and writing. PR: data collection and data analysis. AB: writing. JE: conception, study design, and writing. All authors contributed to the article and approved the submitted version.

## Conflict of Interest

The authors declare that the research was conducted in the absence of any commercial or financial relationships that could be construed as a potential conflict of interest.

## Publisher's Note

All claims expressed in this article are solely those of the authors and do not necessarily represent those of their affiliated organizations, or those of the publisher, the editors and the reviewers. Any product that may be evaluated in this article, or claim that may be made by its manufacturer, is not guaranteed or endorsed by the publisher.
